# Supplementary figures and images for: Presymptomatic microRNA-based biomarker signatures for the prognosis of localized radiation injury in mice
Source: PLoS One. 2025 Aug 8;20(8):e0329737. doi: 10.1371/journal.pone.0329737 (PMC12333984; doi:10.1371/journal.pone.0329737)

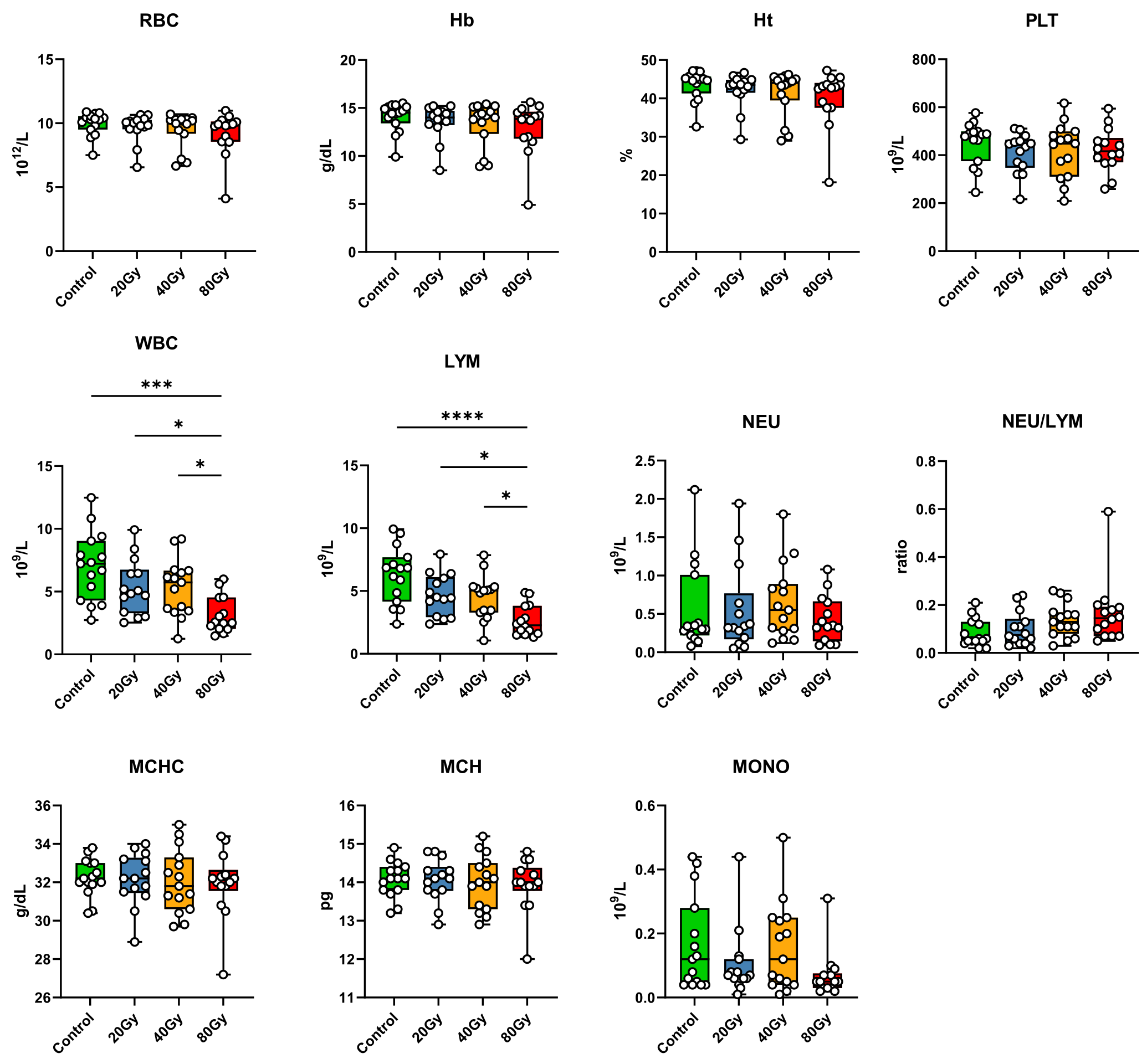

Supplement: S1 Fig — Each animal is represented (n = 13–15/group). Kruskal-Wallis analysis, * P < 0.05; *** P < 0.001; **** P < 0.0001. RBC, red blood cells; Hb, hemoglobin; Ht, hematocrit; PLT, platelet; WBC, white blood cells; NEU, neutrophils; LYM, lymphocytes; MCHC, Mean corpuscular hemoglobin concentration; MCH, mean corpuscular hemoglobin; MONO, monocytes. (TIF) [file pone.0329737.s001.tif]

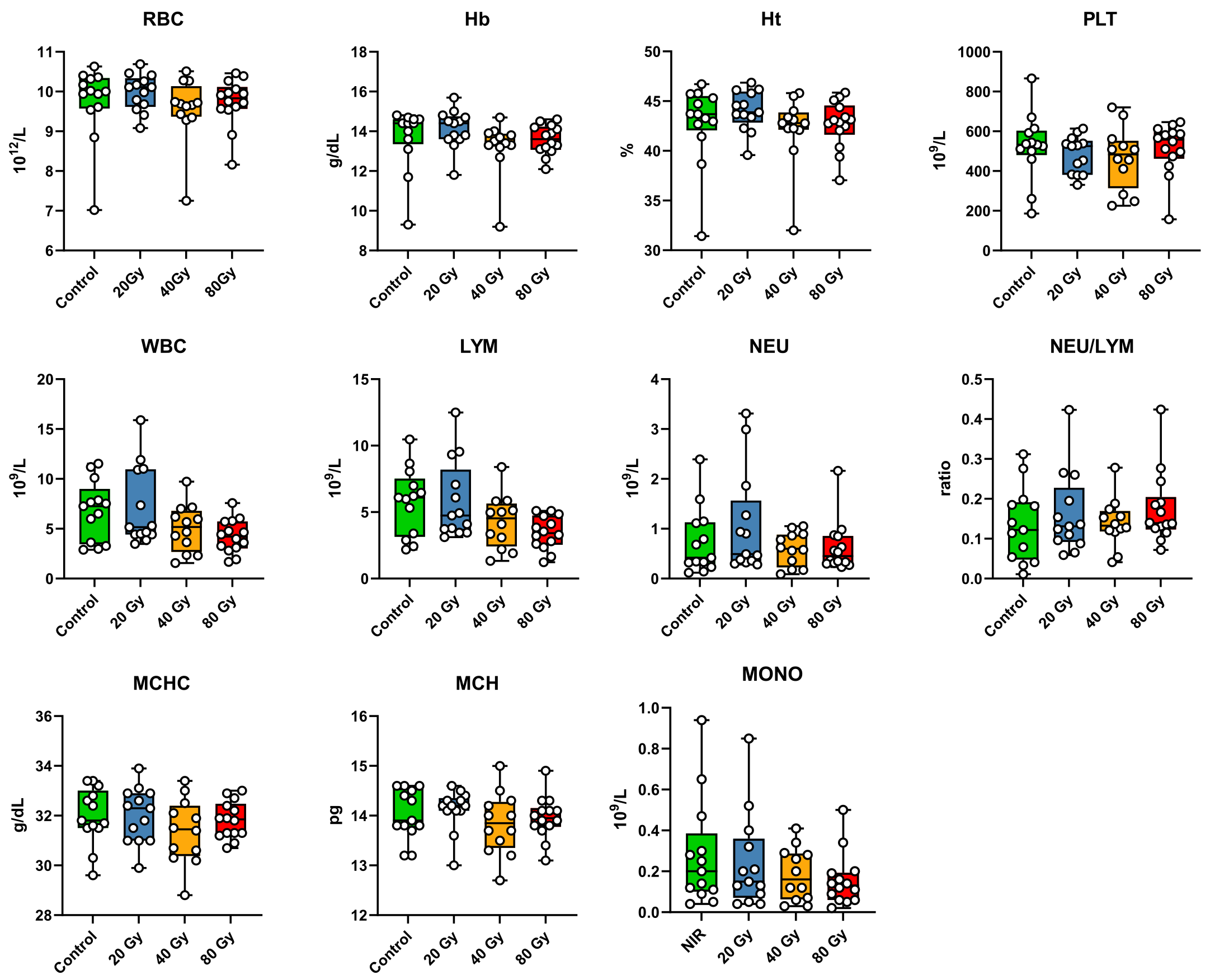

Supplement: S2 Fig — Each animal is represented (n = 12–15/group). Kruskal-Wallis analysis. (TIF) [file pone.0329737.s002.tif]
